# Modified solid lipid nanoparticles encapsulated with Amphotericin B and Paromomycin: an effective oral combination against experimental murine visceral leishmaniasis

**DOI:** 10.1038/s41598-020-69276-5

**Published:** 2020-07-22

**Authors:** Shabi Parvez, Ganesh Yadagiri, Mallikarjuna Rao Gedda, Aakriti Singh, Om Prakash Singh, Anurag Verma, Shyam Sundar, Shyam Lal Mudavath

**Affiliations:** 1 Infectious Disease Biology Laboratory, Chemical Biology Unit, Institute of Nano Science and Technology, Habitat Centre, Phase 10, Sector 64, Mohali, Punjab 160062 India; 2 Department of Biochemistry, Institute of Science, Banaras Hindu University, Varanasi, Uttar Pradesh 221005 India; 3 Infectious Disease Research Laboratory, Department of Medicine, Institute of Medical Sciences, Banaras Hindu University, Varanasi, Uttar Pradesh 221005 India; 4 School of Pharmaceutical Sciences, IFTM University, Moradabad, Uttar Pradesh 244001 India

**Keywords:** Infectious diseases, Nanomedicine

## Abstract

The development of an effective oral therapeutics is an immediate need for the control and elimination of visceral leishmaniasis (VL). We exemplify the preparation and optimization of 2-hydroxypropyl-β-cyclodextrin (HPCD) modified solid lipid nanoparticles (SLNs) based oral combinational cargo system of Amphotericin B (AmB) and Paromomycin (PM) against murine VL. The emulsion solvent evaporation method was employed to prepare HPCD modified dual drug-loaded solid lipid nanoparticles (m-DDSLNs). The optimized formulations have a mean particle size of 141 ± 3.2 nm, a polydispersity index of 0.248 ± 0.11 and entrapment efficiency for AmB and PM was found to be 96% and 90% respectively. The morphology of m-DDSLNs was confirmed by scanning electron microscopy and transmission electron microscopy. The developed formulations revealed a sustained drug release profile upto 57% (AmB) and 21.5% (PM) within 72 h and were stable at both 4 °C and 25 °C during short term stability studies performed for 2 months. Confocal laser scanning microscopy confirmed complete cellular internalization of SLNs within 24 h of incubation. In vitro cytotoxicity study against J774A.1 macrophage cells confirmed the safety and biocompatibility of the developed formulations. Further, m-DDSLNs did not induce any hepatic/renal toxicities in Swiss albino mice. The in vitro simulated study was performed to check the stability in simulated gastric fluids and simulated intestinal fluids and the release was found almost negligible. The in vitro anti-leishmanial activity of m-DDSLNs (1 µg/ml) has shown a maximum percentage of inhibition (96.22%) on intra-cellular amastigote growth of *L. donovani*. m-DDSLNs (20 mg/kg × 5 days, *p.o.*) has significantly (*P* < 0.01) reduced the liver parasite burden as compared to miltefosine (3 mg/kg × 5 days, *p.o.*) in *L. donovani*-infected BALB/c mice. This work suggests that the superiority of as-prepared m-DDSLNs as a promising approach towards the oral delivery of anti-leishmanial drugs.

## Introduction

Visceral leishmaniasis (VL), also known as Kala-Azar is the most severe form of leishmaniasis, a neglected tropical disease caused by the protozoan parasite *Leishmania donovani,* The disease is transmitted to human host by the bite of an infected female haemo-flagellate sand fly^1^. According to WHO, 0.5–0.9 million new VL cases are reported every year, > 95% of which occur in ten countries Bangladesh, Brazil, China, Ethiopia, India, Kenya, Nepal, Somalia, South Sudan and Sudan^2^. These figures do not reflect the true social impact of this disease because VL has a focal distribution that is devastating to the affected communities. Clinically, VL is characterized by a persistent fever, enlargement of spleen and liver, pancytopenia, anemia and weight loss; if untreated, it leads to death of the patient^3^. On the Indian subcontinent, India, Nepal and Bangladesh has set the target for VL elimination as a public health problem as 2020^4^. Early diagnosis, followed by complete treatment, is one of the strategies adopted in this elimination program. However, this disease is also neglected in terms of new drug development since there is little potential for financial return. Currently, few anti-leishmanial drugs are available for the treatment of disease. Although antimonial chemotherapy was the mainstay for VL treatment in the past, parasite resistance against these drugs, especially in the Indian subcontinent, led to the introduction of other drugs such as Amphotericin B (AmB), AmBisome, Miltefosine, and Paromomycin (PM) to treat VL in areas of antimonial drug resistance. However, these drugs are also associated with problems, such as toxicity, high cost, potential development of parasite drug resistance, and prolonged treatment regime^5–7^. Recently, a single dose of AmBisome was found to be sufficient to successfully treat VL with low toxicity and has now been recommended as a choice of treatment in India subcontinent. Importantly, for the drugs like AmBisome and AmB, infusion-related fever, chills and rigor are some of the common side effects with parenteral administration^8–10^. Miltefosine, which is an oral drug, was registered in the Indian subcontinent in 2002 for VL and antimony refractory VL patients but its clinical use was limited because of its teratogenicity^11^. PM (11 mg/kg/day for 3 weeks) has also been recommended drug of choice in *Leishmania donovani*-infected Indian patients^12^. Further, parasite persists in infected individuals even after treatment with these drugs and there is no sterile cure. So, it is necessary to establish the level of parasite burden that is low enough to prevent parasite transmission, but at the same time, maintain concomitant immunity, and this goal may be achieved through nanomodifications^13^. Monotherapy with anti-leishmanials has led to the development of drug-resistant parasites. Therefore, multidrug combination strategies have emerged as a viable option in the treatment of infectious diseases like tuberculosis, leprosy, and malaria. The rationale behind combination therapy is to enhance the therapeutic activity by using drugs having synergistic or additive effects, which can impede drug resistance. Combination therapies have been advantageous because of lower treatment duration, dosage, toxicity and better compliance. Several clinical and pre-clinical studies have shown that the combination of drugs against leishmaniasis is more effective than monotherapy^14^. Combination therapy could enhance therapeutic efficacy for complicated cases, such as HIV-VL co-infected patients, for whom treatment outcomes with monotherapy have been consistently poor^15^. More recent findings have shown a synergistic interaction between AmB and PM in experimental and clinical VL. Co-treatment with PM and AmB has shown a synergetic effect in *leishmania* infected mouse peritoneal macrophages^16^. Clinically, a combination of PM and pentavalent antimonials have also shown protective effects in patients with VL infection in India, Kenya and Sudan^17^.

To overcome the failure of anti-leishmanial drug therapies, the design and development of pertinent drug-carrier systems are of immediate importance. Several drug delivery systems have been developed against VL with enhanced efficacy and reduced toxicity concerns^18–22^. In in vivo*,* the efficacy of the drug is not solely dependent on its physico-chemical properties, but also the carrier system, which could enhance the bioavailability, concede a localized and controlled release of the drug which endow to the explicit requirements of therapy to maximize efficacy, patient safety and compliance^23^. Currently, solid lipid nanoparticles (SLNs) have gained more importance owing to its benefits like affordable cost and safety, than other colloidal nanoparticles. SLNs were introduced in 1991 and consisted of a core of solid lipid with surfactants having a size not more than 1,000 nm^24,25^. The matrix of the lipid core has a significant role in regulating the release pattern and protects the loaded drugs from chemical and enzymatic degradation^26,27^. In SLNs, the matrix is a composition of high melting solid lipids, different from the liposome and emulsion, where the vesicles and droplets are made up of low melting phospholipids and liquid oil, respectively^28^. Lipid-based formulations can decrease drug toxicity and improve bioavailability due to its unique physiological and biodegradable properties^27^.

Oral drug delivery is the most desirable delivery route being non-invasive, convenient and cost-effective. Endogenously, the SLNs are conducive for enhancing the oral uptake of the therapeutic agent by retaining a solubilized state of the drug in the GIT and favors the formation of mixed micelles by inducing the secretion of bile salts and phospholipids. Further, 2-hydroxypropyl-β-cyclodextrin (HPCD) are cyclic oligosaccharides that have been acknowledged in recognition chemistry as molecular hosts capable of including, with a degree of selectivity, water-insoluble guest molecules via non-covalent interactions within their hydrophobic cavity. The understanding of cyclodextrins to enhance oral or dermal absorption of drugs is a well-documented property, principally in the case of poorly water-soluble drugs. This property has been attributed to the increased thermodynamic activity of the drug in the vehicle and/or to enhance the rate of drug dissolution^29–33^.

In this current study, we exemplify the preparation and characterization of HPCD modified SLNs based combinational cargo system of AmB and PM. Employing a combination of in vitro and in vivo experiments, we have shown that HPCD modified SLNs can demonstrate the enhanced anti-leishmanial activity with reduced toxicity when administered orally.

## Materials and methods

### Chemicals

Glyceryl monostearate (GMS), soya lecithin (SL), polyoxyethylene sorbitan monooleate (Tween 80), cellulose dialysis tube (Mol. Weight; 14 kDa), Polyethylene glycol 400 (PEG 400) were obtained from HiMedia Laboratories (India). Trehalose was procured from TCI Chemical, India. Amphotericin B, paromomycin, 4′, 6-diamidino-2-phenylindole (DAPI), fluorenylmethoxycarbonyl chloride (Fmoc-Cl) and fluorescein isothiocyanate isomer I (FITC) were procured from Sigma-Aldrich (U.S.A). Fetal bovine serum (FBS), RPMI 1,640, penicillin–streptomycin, phalloidin-rhodamine, trypsin-ethylene diamine tetra acetic acid (Trypsin–EDTA) and phosphate buffer saline (PBS; pH–7.4), were purchased from Invitrogen (USA). Glass bottom dishes from the ThermoFiscer Scientific (USA), Silicon wafers and Carbon-coated TEM grids from Beta Tech Equipment (India).

### Animals

Male BALB/c mice (20 ± 2 g) and Swiss albino mice (22 ± 2 g) were used for studying anti-leishmanial activity and toxicity profiles, respectively. BALB/c mice were procured from the central animal facility of Central Drug Research Institute, Lucknow and male Swiss albino mice (22 ± 2 g) were procured from the central animal facility, IMS, BHU. All the experiments were performed in accordance with the relevant laws of the Committee for the Purpose of Control and Supervision of Experiments on Animals (CPCSEA), Ministry of Environment and Forests, Government of India. The Central Animal Ethics Committee (CAEC), IMS, BHU (CAEC number Dean/2017/CAEC/269) approved the animal protocols. Animals were kept in cages and maintained 12 h light–dark cycle with standard feed and water ad libitum.

### Parasite and cell line

*Leishmania donovani* (LEM 138) promastigotes were used for in vitro and in vivo anti-leishmanial activity testing. *L. donovani* promastigotes were cultured in M199 medium with 10% heat-inactivated fetal bovine serum (HIFBS), antibiotics (100 U/ml penicillin, 100 µg/ml streptomycin) and maintained at 26 °C BOD incubator. Cultures were passaged twice a week and maintained for not more than 1 month. Macrophage cell line (J774A.1) was procured from NCCS, Pune, India and was cultured in RPMI-1640 supplemented with 10% HIFBS, 100 U/ml penicillin and 100 μg/ml streptomycin at 37 °C and 5% CO_2_ environment.

### Formulation and development

#### Preparation of DDSLNs

Dual drug loaded solid lipid nanoparticles (DDSLNs) were prepared using combination of AmB and PM through emulsion/solvent evaporation method^34,35^ with a slight modification. Briefly, 150 mg of GMS, 40 mg of SL and 50 mg of AmB were dissolved in 5 ml of ethanol at 60 °C and used as an oil phase. The aqueous phase was prepared by adding a stabilizer (polyvinyl alcohol; 0.5% w/v) and PEG 400 (1.5% w/v) along with PM (20 mg) maintained at the same temperature with continuous stirring for obtaining a dispersion. The organic phase was then added to aqueous phase drop wise set at 60 °C under rapid stirring at 1,000 rpm for 3 h. The homogeneous suspension was then dropped into a dispersed phase containing 1% Tween 80 and 1% PEG 400 at 2–4 °C for 4 h to permit hardening of solid lipid nanoparticles (SLNs).

#### Preparation of m-DDSLNs

The modified DDSLNs (m-DDSLNs) were prepared by dispersing 5 ml of DDSLNs in an aqueous phase consisted of dispersion of 2-hydroxypropyl-β-cyclodextrin (2-HPCD) and trehalose in Milli-Q water. Briefly, 1% (w/v) of 2-hydroxypropyl-β-cyclodextrin (2-HPCD) was dispersed in Milli-Q water followed by shaking at 4 °C for 4 h at 50 rpm and trehalose (2% w/v) was used as cryoprotectant^36^. The prepared dispersion was freeze dried according universal step wise freeze drying process in a freeze drier (FDUT 12,003, Republic of Korea). The dispersion was kept at − 80 °C for 24 h and subsequently the formulations were transferred to the freeze drier. The suspension was centrifuged at 11,000 rpm for half an hour and washed with Milli-Q water twice to ensure the removal of organic solvents completely. The freshly prepared SLNs dispersion was then freeze-dried (FDUT-12003, Republic of Korea) to obtain m-DDSLNs in powdered form and further stored at 4 °C until use. Plain SLNs (PSLNs) were also prepared using the same method without the addition of drugs.

### Measurement of particle size and zeta potential

The particle size (PS) and zeta potential (ZP) of nanoparticles were assessed by quasi elastic-light scattering analysis using a Zetasizer (Malvern, Nano ZS90, UK) at 25 °C under detection angle of 90° for PS and polydispersity index (PDI) and 120° for ZP and all the experiments were run in triplicate. Briefly, lyophilized samples were diluted (1:50) with milli-Q water and placed in polystyrene disposable cuvettes (Model-DTS0012; Malvern) for the measurement of size and in disposable folded capillary cells (Model-DTS1070; Malvern) for the measurement of surface charge. All samples were appropriately diluted in Milli-Q water for optimum kilo counts per seconds (Kcps). All the experiments were performed in triplicate.

### Entrapment efficiency and drug loading

The entrapment efficiency (EE) and drug loading (DL) was determined by slight modifications of a previously reported indirect method^37^. The dispersion of nanoparticles in Milli-Q was centrifuged at 11,000 rpm for 30 min and the supernatant was isolated. The percentage of the drug (AmB) in the supernatant was estimated by UV–visible spectrophotometer (Shimadzu, UV-2600) at 405 nm.

The EE and DL was determined by using following equations

1 $${\text{EE}}\,\left( \% \right) \, = {\text{ W}}_{{\text{t}}} - {\text{ W}}_{{\text{s}}} {\text{/W}}_{{\text{t}}} *100$$

2 $${\text{DL}}\,\left( \% \right) \, = {\text{ W}}_{{\text{t}}} - {\text{ W}}_{{\text{s}}} {\text{/W}}_{{\text{l}}} + {\text{ W}}_{{\text{t}}} - {\text{ W}}_{{\text{s}}} *{1}00$$

W_t_, the total amount of drug added; W_s_, the amount of drug present in the supernatant; W_l_, the amount of the lipid added.

As PM is inactive for UV–Visible spectroscopy the EE (%) of PM was calculated by fluorescence spectroscopy. Firstly, Fmoc-Cl-PM was synthesized by the solution-phase process. Briefly, Fmoc-Cl (4 mM solution) was taken in acetonitrile and added to PM solution (0.5 mM) in borate buffer (0.5 M; pH 8.1), stirred for 15 min in dark and later, ethyl acetate was added to extract the reaction mixture for the preparation of DDSLNs. The suspension was then centrifuged at 11,000 rpm for 30 min and the supernatant was analyzed using an F-4600 spectrophotometer (Hitachi, Japan) at 315 nm up on excitation at 260 nm^38^.

### Characterization of nanoparticles

#### Electron microscopy

The morphology of m-DDSLNs was determined by scanning electron microscopy (SEM; IT300, JEOL 6,400, JEOL USA, Peabody, MA.) and the transmission electronic microscopy (TEM; JEOL 2,100, (Tokyo, Japan)). Briefly, freeze dried m-DDSLNs were dispersed in Milli-Q water to prepare 100 µg/ml dispersion. For SEM, 10 µl of this dispersion was drop-casted, air dried followed by sputter coating with colloidal gold under vaccum using JEC-300FC analysed at 8 kV.

For TEM, 10 µl of diluted formulation was placed on copper grid, air dried at room temperature followed by negative staining with 2% w/v phosphotungstic acid (PTA) and washing with Milli-Q water. The samples were then vacuum dried, and images were obtained at an accelerating voltage of 200 kV having lanthanum hexaboride (LaB6) filament. TEM micrographs were captured using Gatan camera software.

#### Fourier transform infrared spectroscopy (FTIR)

The determination of drug-excipient interactions was evaluated by ATR-FTIR Spectroscopy (Bruker vertex 70v spectrophotometer). The scanning range was set at 400–4,000 cm^−1^ for each spectrum at the resolution of 4 cm^−1^ and 64 scans.

#### Powder X-ray diffraction (PXRD)

X-ray diffractogram of AmB, PM, PSLNs, DDSLNs, m-DDSLNs was obtained using an X-ray diffractometer (Bruker, D8 Advance X-ray Diffractometer) equipped with a Cu-Kα radiation source (λ = 1.541 Å). The instrument was fixed at 40 kV & 25 mA and the diffraction angle (2θ) was measured from 10° to 60°.

### Storage stability studies of nanoparticles

Temperature as well as time-dependent stability studies, were carried out using DDSLNs and m-DDSLNs at two different temperatures (25 ± 2 °C and 4 ± 2 °C) for 2 months. All the samples were examined for mean particle size, PDI and zeta potential^39^ and samples were stored in a glass vial with air tight rubber caps at 25 °C and 4 °C for a period of 60 days. Every month, the particle size, PDI and Zeta potential of the nanoparticles was evaluated as reported previously^40^.

### In vitro simulated gastrointestinal fluid stability studies

To access the stability of SLNs, formulations were placed in the HPMC capsule and kept in dissolution basket containing 500 ml medium [simulated gastric fluid (SGF 1.6 pH) and simulated intestinal fluids (SIF pH 6.5)] at 37 ± 0.5 °C under continuous stirring at 50 rpm. The simulated gastric and intestinal fluids were prepared as per composition and procedure reported by Klein et al.^41^. The SGF was prepared by adding lecithin (20 µM), pepsin (0.1 mg/ml), sodium chloride (34.2 mM), sodium taurocholate (80 µM), HCl conc. (q.s. pH 1.2) in 500 ml deionized water and SIF was prepared by adding sodium taurocholate (3 mM), lecithin (0.75 mM), sodium di-hydrogen phosphate (3.43 gm), sodium chloride (6.18 gm), sodium hydroxide (*q.s.* pH 6.5) in 500 ml deionised water.

### In vitro drug release study

In vitro drug release was evaluated by using a dialysis bag method. Nanoparticles were placed in dialysis bag (soaked overnight in double-distilled water) and sealed with clamps. The bag was placed in 250 ml of PBS (pH 7.4) having 1% Tween 80 and stirred continuously at 100 rpm maintained at 37 ± 1 °C. The Tween 80 was added to the release medium as AmB solubility issue was observed in the absence of Tween 80 that lead to error in detection of drug by UV–Visible spectroscopy. Aliquots were then withdrawn at a fixed time interval and replaced with a fresh buffer to maintain sink condition. Samples withdrawn at a particular interval were analyzed using a UV–visible spectrophotometer at 413 nm (Shimadzu UV-2600)^42^ and microplate reader (Infinite 200 PRO microplate plate reader). The cumulative percentage of drug released was calculated and the mean was used in data analysis.

### In vitro cytotoxicity assay

Cytotoxicity on m-DDSLNs, PSLNs, AmB and PM was evaluated by using MTT assay on the J774A.1 macrophage cell line. Briefly, J774A.1 macrophage (5 × 10^4^ cells/well) were seeded into 96 well plate and incubated for 24 h at 37 °C & 5% CO_2_ environment allowing the cells to adhere. Then, cells were treated with varying concentrations (0.1, 1, 5, 10 μg/ml) of equivalent drug concentrations of different formulations and pure drugs for 24 h while untreated cells served as control and 20 μl of MTT reagent (5 mg/ml) was added to each well followed by incubation for 4 h. DMSO (100 μl) was added to solubilize formazan crystals. The optical density (OD) was taken at 570 nm by using a microplate reader (Infinite 200 PRO microplate plate reader). Each experiment was performed in triplicate.

### Macrophage uptake study

FITC-loaded SLNs (FITC-SLNs) were prepared by slight modifications of a previously reported emulsion/solvent evaporation method. Briefly, J774.A.1 cells were seeded into glass bottom dishes (Nunc, Thermo Scientific, United States) at an initial cell density of 5 × 10^4^ cells per dish and incubated in 5% CO_2_ environment at 37 °C for 24 h. The cells were treated with 50 µg/ml of FITC-SLNs and incubated for 6 h, 12 h and 24 h. The cells were washed with 1 × PBS to remove any non-phagocytosed FITC-SLNs. The cells were then fixed with 4% paraformaldehyde for 20 min followed by washing and then staining with DAPI for 3 min. Further, the cells were incubated with rhodamine-phalloidin for half an hour to stain the cell membrane^43^. Lastly, the fixed cells were washed and observed under a confocal laser scanning microscope (CLSM, LSM 880 NLO, Carl Zeiss, Germany).

### In vitro anti-leishmanial activity of m-DDSLNs against intra-cellular amastigotes of *L. donovani*

In vitro anti-leishmanial activity of m-DDSLNs were tested against intra-cellular amastigotes of the *leishmania* parasite*.* Briefly, macrophage cells (J774A.1) (2.5 × 10^4^ cells/ml) were resuspended in complete RPMI-1640 and seeded in eight well Lab Teck tissue culture slides (Nunc, USA) and incubated at 37 °C and 5% CO_2_ environment for 2 h for macrophage adherence. The adherent macrophages were washed (3 ×) with pre-warmed incomplete RPMI-1640 and infected with metacyclic promastigotes in 1:10 ratio and incubated at 37 °C and 5% CO_2_ environment for 12 h. Non-phagocytosed promastigotes were discarded by simple exchange of medium and the infected macrophages were incubated with and without having test/reference drugs (m-DDSLN, AmB SLN, AmBisome, AmB and PM) in different concentrations (0.1–1 µg/ml) in complete RPMI-1640 at 37 °C and 5% CO_2_-air atmosphere for 72 h. The infected macrophages were washed with PBS and stained with Wright’s stain to assess the intra-cellular amastigote growth and intra-cellular amastigotes were monitored by counting at least 100 cells per slide under oil immersion lens microscope (100 ×)^44^.

The following formula calculated percentage inhibition of amastigote replication:

$${\text{PI }} = \, 100 - \left( {\text{AT/AC}} \right) \, \times 100$$

PI, percentage inhibition of amastigote multiplication; AT, actual number of amastigotes in treated samples/100 macrophages; AC, actual number of amastigotes in control samples/100 macrophages.

### In vivo toxicity study

#### Sub-acute toxicity study

Developed formulations and standard drugs [m-DDSLNs (*p.o.*), AmB (*i.v.*), PM (*i.m*)] were administered to Swiss albino mice (n = 6) for 14 days, daily at a dose of 20 mg/kg. Blood samples were collected on 14 days of post-treatment and determined the serum creatinine, blood urea nitrogen (BUN), serum glutamic-oxaloacetic transaminase (SGOT) and serum glutamic pyruvic transaminase (SGPT) by using commercially available kits.

### In vivo anti-leishmanial activity of m-DDSLNs in *L. donovani*-infected BALB/c mice

BALB/c mice were infected with 1 × 10^7^ *L. donovani* promastigotes on day 0. Treatment was started with test (m-DDSLNs) and reference (miltefosine) drugs on day 7 to day 11 of post-infection (5 consecutive days). All the animals in each group were sacrificed on day 14 of post-infection; Liver and spleens were collected aseptically and impression smears were prepared for parasitological observation^45^. The course of infection was monitored by examination of Wright’s stained liver imprints under oil immersion lens microscope (100 ×). The parasite burden was quantified as the number of amastigotes/500 host cell nuclei or by calculating Leishman-Donovan Units (LDU).

$${\text{LDU }} = \, \left( {{\text{Number}}\,{\text{of}}\,{\text{amastigotes/5}}00\,{\text{host}}\,{\text{cell}}\,{\text{nuclei}}} \right) \, \times {\text{ organ}}\,{\text{weight}}\,\left( {{\text{mg}}} \right)$$

Percentage inhibition of liver parasite burden and percentage suppression of parasite replication were calculated.

$${\text{Formula:}}\quad {\text{PI}} = \left( {{\text{PP}} - {\text{PT/PP}}} \right) \, \times { 1}00$$

PP, No. of amastigotes/500 host cell nuclei in the liver before treatment; PT, No. of amastigotes/500 host cell nuclei in the liver after treatment;PC, No. of amastigotes/500 host cell nuclei in the liver after treatment in the control group.

## Results and discussion

### Preparation and characterization of modified dual drug loaded solid lipid nanoparticles

**Figure 1:**
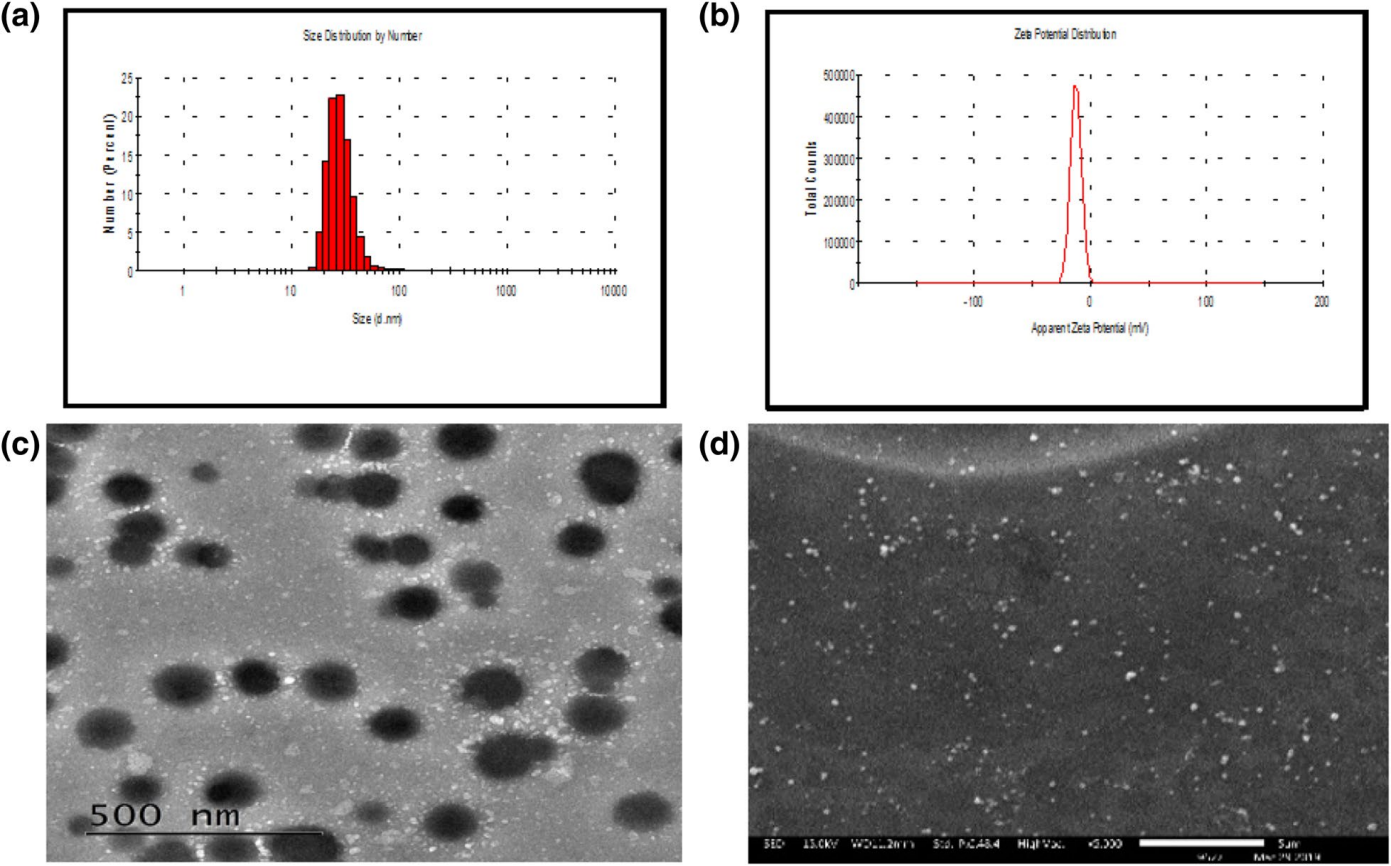
(**a**) Mean particle size (nm) of m-DDSLNs. (**b**) zeta potential (mV) of m-DDSLNs. (**c**) TEM image of m-DDSLNs. (**d**) SEM image of m-DDSLNs.

Plain (PSLNs), unmodified (DDSLNs) and modified dual drug loaded solid lipid nanoparticles (m-DDSLNs) were successfully developed using emulsification solvent evaporation method, a well-established and extensively used method^46^. Table 1 lists the mean PS, PDI, ZP, %EE and drug loading (%DL) of PSLNs, DDSLNs and m-DDSLNs. PSLNs were prepared by using PEG 400: PVA (1.5:1) with 150 mg GMS, showed mean PS of 38.16 ± 0.51 nm having PDI of 0.40 ± 0.21. The addition of higher quantity surfactant, PEG 400 can act as a hydrophilic shield and therefore lowers the surface tension of particles, ultimately reducing the particle size. The particle size was increased to 141 ± 3.2 on loading of AmB and PM in DDSLNs. Further, after HPCD modification the mean particle size was found to be 164.3 ± 17.6 nm (Fig. 1a) with ZP of − 14.7 ± 3.40 mV (Fig. 1b).

**Table 1:** Characteristics of PSLNs, DDSLNs and m-DDSLNs.

Formulations	Mean particle size (nm)	PDI	Zeta potential (mV)	AmB		PM	
				% EE	%DL	% EE	%DL
PSLNs	38.16 ± 0.51	0.40 ± 0.21	− 8.44 ± 0.20	–	–	–	–
DDSLNs	141 ± 3.2	0.248 ± 0.11	− 12.3 ± 1.89	95.80 ± 0.27	24.3 ± 0.37	90.50 ± 0.28	9.2 ± 0.56
m-DDSLNs	164.3 ± 17.6	0.392 ± 0.18	− 14.7 ± 3.40	95.86 ± 0.10	24.0 ± 0.27	90.3 ± 0.28	10.4 ± 0.02

Results are presented as mean ± standard deviation, (n = 3).

Entrapment efficiency and drug loading of m-DDSLNs was found by UV–Vis and fluorescence spectroscopies for AmB and PM, respectively. The %EE for AmB and PM in m-DDSLNs was found to 96% and 90% while the loading capacity was calculated as 24% (AmB) and 10.5% (PM). The %EE increases with hydrophobicity of drug due to their strong interaction with lipid matrix. AmB present both in the bilayers as well as in internal solid lipid core^47^. Also, the higher %EE of AmB is attributed to its lipophilic nature^48^.

### Physicochemical characterization

#### Electron microscopy

SEM can produce images that are good representations of the three dimensional shape of the sample, whereas, a TEM can achieve better than 50 pm resolution and magnifications of up to about 10,000,000 ×. Generally, the image resolution of an SEM is at least an order of magnitude poorer than that of a TEM.

Both TEM and SEM studies were performed to confirm the morphological characteristics of nanoparticles. TEM demonstrated the spherical shape of m-DDSLNs and mean particle size was found to be 136.90 ± 8.89 nm (Fig. 1c). In SEM, m-DDSLNs were observed as discrete, spherical particles which are regular in shape, with particle size of 140.2 ± 10.15 nm (Fig. 1d). A difference was observed in the measurement of particles size, between by QELS and electron microscopy. As nanoparticles in QELS were measured in solution and thus has solvent layer around the nanoparticles, it gives hydrodynamic diameter while for electron microscopy, nanoparticles measured in dried form.

#### FTIR

**Figure 2:**
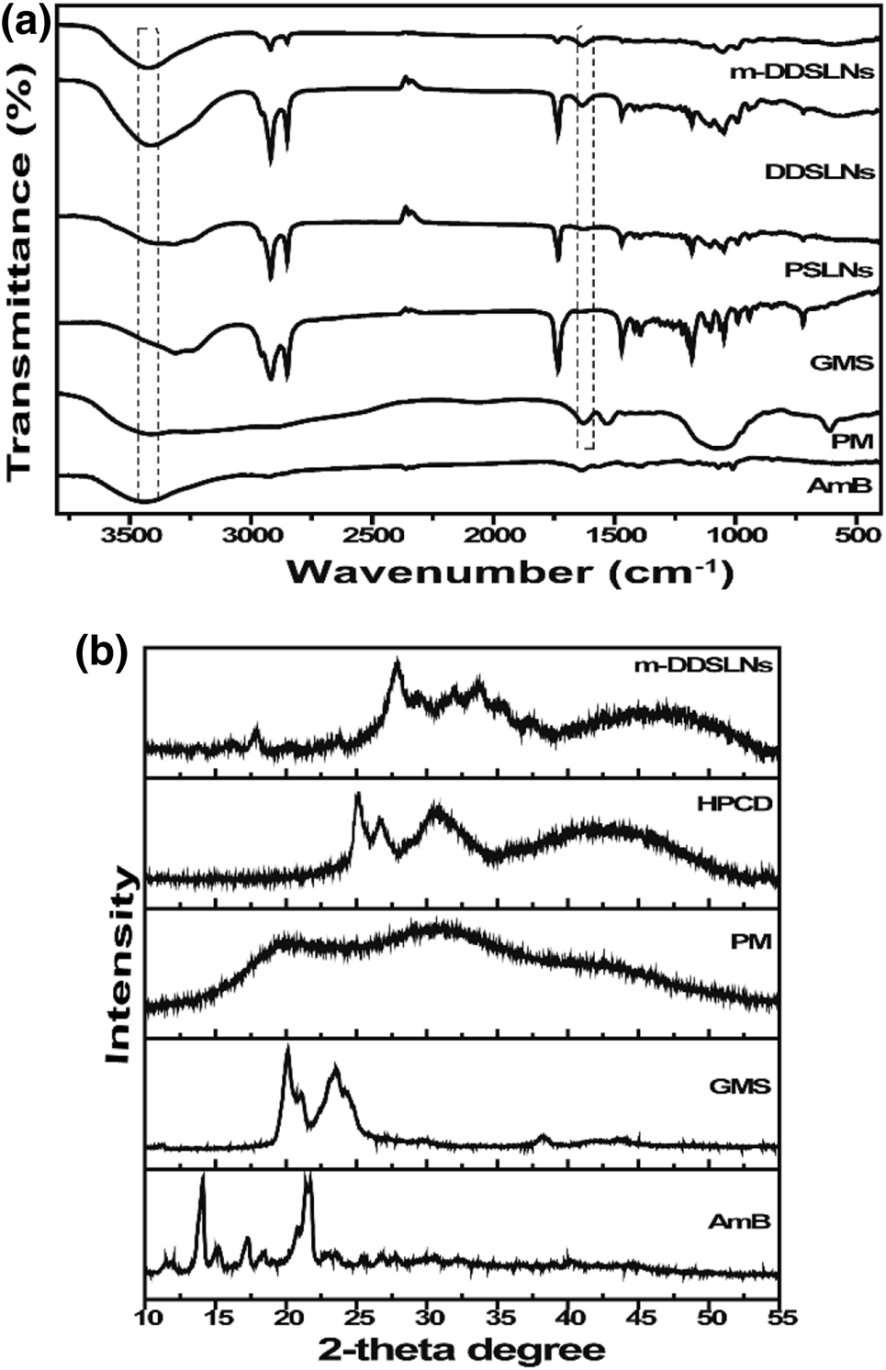
(**a**) FTIR spectra for AmB, PM, GMS, PSLNs, DDSLNs, m-DDSLNs. (**b**) XRD pattern for GMS, PM, AmB, HPCD, m-DDSLNs.

Figure 2a represents the FTIR spectra of AmB, PM, GMS, PSLNs, DDSLNs and m-DDSLNs. It was observed that the absorption band for GMS was present at 1,730 cm^−1^ corresponds to the C=O stretching band of fatty acid ester. The peak at 1,166 cm^−1^ indicates C–O–C stretching and a characteristic band at 3,300 cm^−1^ is due to O–H stretching in the glycerol moiety. C–H stretching bands (718 & 1,457 cm^−1^) denoted the aliphatic side chain. No considerable differences in position, shape as well as the intensity of peaks related to GMS, was detected in comparison with PSLNs, DDSLNs spectra, indicating the solidification of lipid in the nanoparticles as a matrix having a similar arrangement to that of the original components^49^. The FTIR spectra of AmB showed distinctive peaks such as –OH stretch (3,438 cm^−1^), C=C bond (1558 cm^−1^) and NH_2_ in-plane band (1634 cm^−1^) shown in Fig. 2a C–O stretching (1,368.09 cm^−1^), C–H (757.14 cm^−1^) and N–H (669.14 cm^−1^)^39^. In the FTIR spectrum of DDSLN, peaks corresponding to AmB are either absent or broadened, implying the entrapment of drug in the matrix of the lipid core^50^, as shown in Fig. 2a. No additional peaks were observed in the FTIR spectra of m-DDSLNs, suggesting no interaction on surface modification of DDSLNs. The FTIR spectrum also showed the characteristic peaks of PM at 3,408 cm^−1^ (NH_2_ amine group), 1534 cm^−1^ (CH_2_ bending), 1632 cm^−1^ (N–H bending along with C–N stretch) and 1,027 cm^−1^ (C–O–C) stretch are present in DDSLNs^51^.

### P-XRD studies

The P-XRD spectra of GMS, PM, AmB, HPCD, m-DDSLNs shown in Fig. 2b. AmB exhibited a sharp peak at 2θ scattered angle of 14.1°,15.1° and 21.78° indicating its crystallinity and no intensified peaks were observed for PM signifying its amorphous nature. HPCD spectra showed sharp peaks at 19.1°, 20° as well as some broad peaks between 21° and 47°. Sharp and broad peaks were observed in the XRD spectra of m-DDSLNs due to both AmB and HPCD. In m-DDSLNs spectra Peaks present at 13.9°, 15.1° showing the presence of AmB. m-DDSLNs spectra were showing very similar XRD patterns like HPCD spectra revealing its presence on the surface of m-DDSLNs. Diffraction patterns of SLNs formulations (PSLNs and m-DDSLNs) were broader and weaker than the bulk lipid i.e. GMS, due to partial recrystallization and transformation. The less ordered and amorphous nature of m-DDSLNs might contribute to the higher drug loading^52^.

### Stability studies

**Figure 3:**
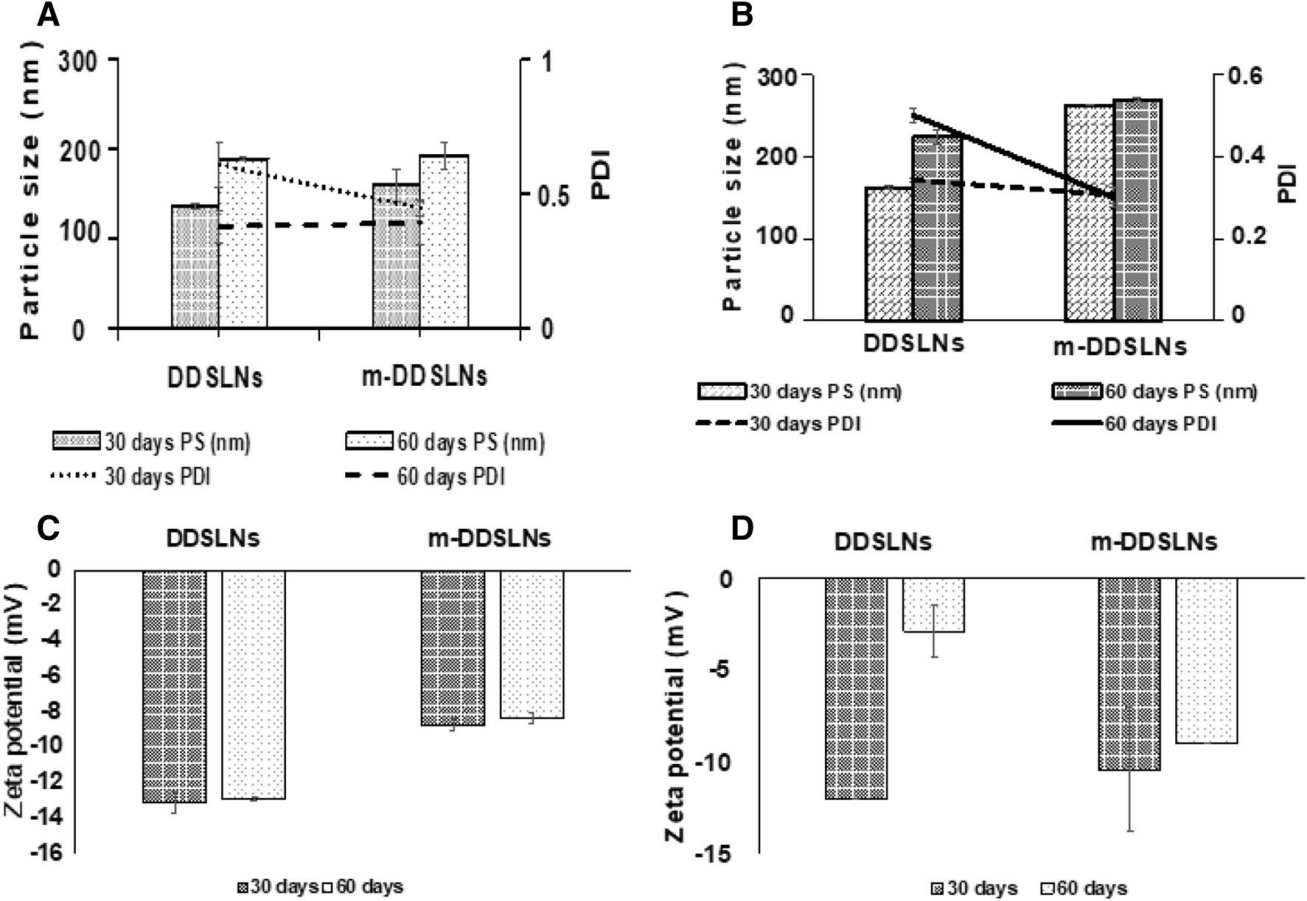
(**a**) Temperature dependent stability at 4 °C for 60 days. (**b**) Temperature dependent stability at 25 °C for 60 days. (**c**) Zeta potential (mV) at 4 °C for 60 days. (**d**) Zeta potential (mV) at 25 °C for 60 day. Results are presented as mean ± standard deviation, (n = 3).

The physical stability of the prepared SLNs was evaluated at 4 °C and 25 °C for 60 days by particle size measurements (Fig. 3). The stability of SLNs during storage conditions is a critical problem in the drug delivery process and may lead to a change in particle size, polymorphism and crystallinity^53^. Therefore, in the present study we tried to evaluate the effect of temperature for 2 months at 25 °C and 4 °C. The mean PS (nm) and PDI of m-DDSLNs were found to increase to 192 ± 15 nm), 0.39 ± 0.08 respectively at 4 °C further at 25 °C size increased to 269.1 ± 2.65 with PDI of 0.30 ± 0.02. However, the mean PS and PDI for DDSLNs were observed to be more when compared to m-DDSLNs (Fig. 3). It was observed that the variation of stability parameters at 4 °C were lower as compared to 25 °C, revealing the stability of SLNs at lower temperatures while changing the temperature towards higher verge, ascribed to particles aggregation might be due to lipid matrix degradation or deformation. Wiedenmann et al. reported that SLNs stabilized by protein, were able to withstand the heat treatment more as compared to Tween 20 stabilized SLNs and found no emulsion coalesce^54^. Researchers have also investigated the stability of ClAlPc/SLNs and unloaded SLNs for 24 months at 4 °C by observing the size, PDI, and ZP^55,56^.

### Simulated gastrointestinal fluid stability study, in vitro

**Figure 4:**
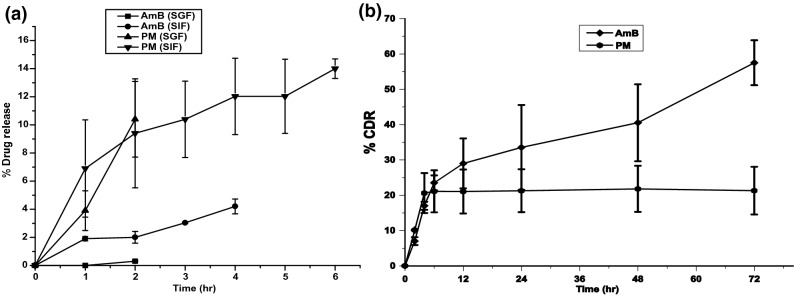
(**a**) In vitro release study in simulated gastric fluids (pH 1.6) and simulated intestinal fluids (pH 6.5) of m-DDSLNs. (**b**) In vitro release study of m-DDSLNs at pH 7.4. Results are presented as mean ± standard deviation (n = 3).

It was anticipated to develop a targeted drug delivery system to specific internal organs (liver and spleen) with minimum drug release in GIT. Drug release studies were performed in media with acidic (pH 1.6) and alkaline (pH 6.5) conditions to determine the stability of SLNs while their transit through the gastrointestinal system for stable administration of the SLNs orally. Drug release for both the drugs was found less than 20%. HPCD coating onto SLNs significantly reduced the drug release in simulated gastrointestinal fluid. The AmB release from the lyophilized m-DDSLNs was studied and it was found that the release was 0.33% (up to 2 h) in SGF and 4.2% (for 4 h) in SIF. While, for PM it was 10% (in 2 h) in SGF and 14% (for 6 h) in SIF as shown in Fig. 4a. It was assumed that the drugs present on the surface of nanoparticles (which is loosely bound), released in the simulated gastric fluids. The presence of lipases in GIT is responsible for lipid matrix degradation and may pose a major hurdle on drug release from SLNs. Interestingly, it was found that HPCD prevents the degradation of insulin while in transit through the GIT^57^. Also, Gould et al*.* reported that HPCD was endured very well in the animal species like mice rats, dogs^58^. Under extreme conditions like the treatment of strong acid and base, HPCD enhanced the stability of insulin^59^. Our study also highlights the role of HPCD in preventing the degradation of m-DDSLNs in GIT, besides preventing its faster release due to lipid erosion under the effect of fluid and enzymes present in the intestinal tract. Further, the in vitro results can be corroborated with further in vivo studies in the murine experimental VL model. Thus, paving the way for the development of a promising oral drug delivery system for more effective therapeutics against VL.

### In vitro drug release study

The in vitro drug release exhibited a biphasic pattern as shown in Fig. 4b which is represented as cumulative % release of AmB (57%) and PM (21.5%) from m-DDSLNs upto 72 h and the initial burst release (17% AmB, 20% PM) upto 4 h is contributed to the presence of the drug on the surface of nanoparticles^60^ which was followed with slow, extended and sustained release up to 72 h. This type of release pattern is commonly present in matrix-based systems where the drug release mainly occurs by spreading across the matrix of the lipid or/and biodegradation as well as surface degradation of the matrix^61^. The lipid barrier or matrix, might be maintaining the release up to 72 h (57%) by restricting the entry of medium and inhibited the faster immobilization of the AmB from the GMS and lecithin core and so, extended the release. The role of SLNs in the biphasic release pattern of the drugs was well documented in the earlier findings^62–64^. Although, it was observed that the release of PM was smaller as compared to AmB. Hydrophilic interaction might be present between hydrophilic head of surfactant, hydrophilic fragment of HPCD as well as PM.

### Cytotoxicity study

**Figure 5:**
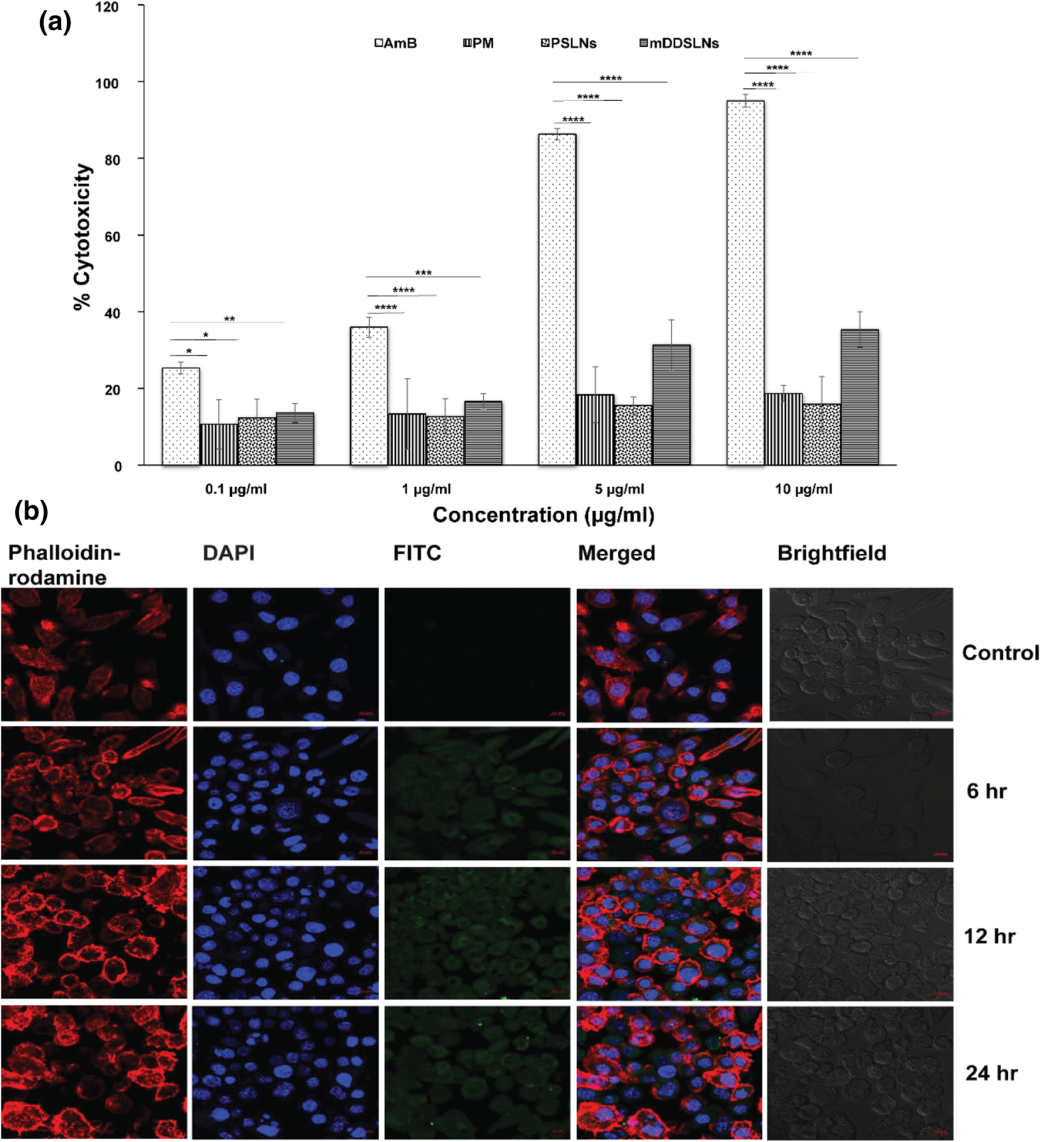
(**a**) % cytotoxicity of macrophage cells (J774A.1) treated with different concentrations 0.1, 1, 5 and 10 µg/ml of AmB, PM, PSLNs, m-DDSLNs. Results are presented as mean ± standard deviation (n = 3). Statistical significance was assessed using two way ANOVA followed by Student's t-test (*P < 0.05 and **P < 0.01, ***P < 0.001, ****P < 0.0001) (**b**) Confocal microscopy images of J774.A.1 cells upto 24 h incubation at 37 °C with FITC-SLNs. *FITC* fluorescein isothiocyanate, *hr* hours.

The cytotoxicity data of the developed formulations m-DDSLNs, PSLNs, AmB and PM in Fig. 5a suggests the developed SLNs are more cyto-biocompatible when compared to the conventional AmB. The cytotoxicity was found to be less than 20% for PSLNs at all the concentration, which confirmed the safety of excipients^65^ used for formulation development. At higher concentrations the cytotoxicity of m-DDSLNs were found to slightly increased but it was significantly (*P* < 0.0001) lesser than AmB, a possible explanation could be controlled release of drugs from the lipid matrix of SLNs. In conformity, with the drug release study as AmB %CDR was found upto 57% in 24 h, might be the reason for toxicity at higher concentration.

### Macrophage uptake study

Cellular uptake study was performed to evaluate the uptake of FITC loaded SLNs by the macrophages up to 24 h and the confocal image suggests complete internalization of FITC labelled SLNs in a time-dependent manner (Fig. 5b). HPCD modification enhanced the uptake of SLNs by the macrophages where *leishmania* amastigotes dwell amply. Previous workers have also shown that HPCD modified paclitaxel-loaded SLNs had enhanced cellular uptake in MCF-7 cells^66^. Also, it was found that HPCD could be used for enhanced transport of insulin across Caco-2 cells monolayer^67^. HPCD could also play an important role in inhibition of p-gp efflux pump, which in turn leads to enhancement of oral bioavailability and cellular uptake. Interestingly, cyclodextrins can act as an absorption enhancer, as well as effect the epithelial barrier properties^30^. Better internalization of FITC-SLNs is precisely associated with the enhanced in vitro and in vivo activity.

### In vitro anti-leishmanial activity of m-DDSLNs against *L. donovani*-infected J774A.1 macrophage

**Figure 6:**
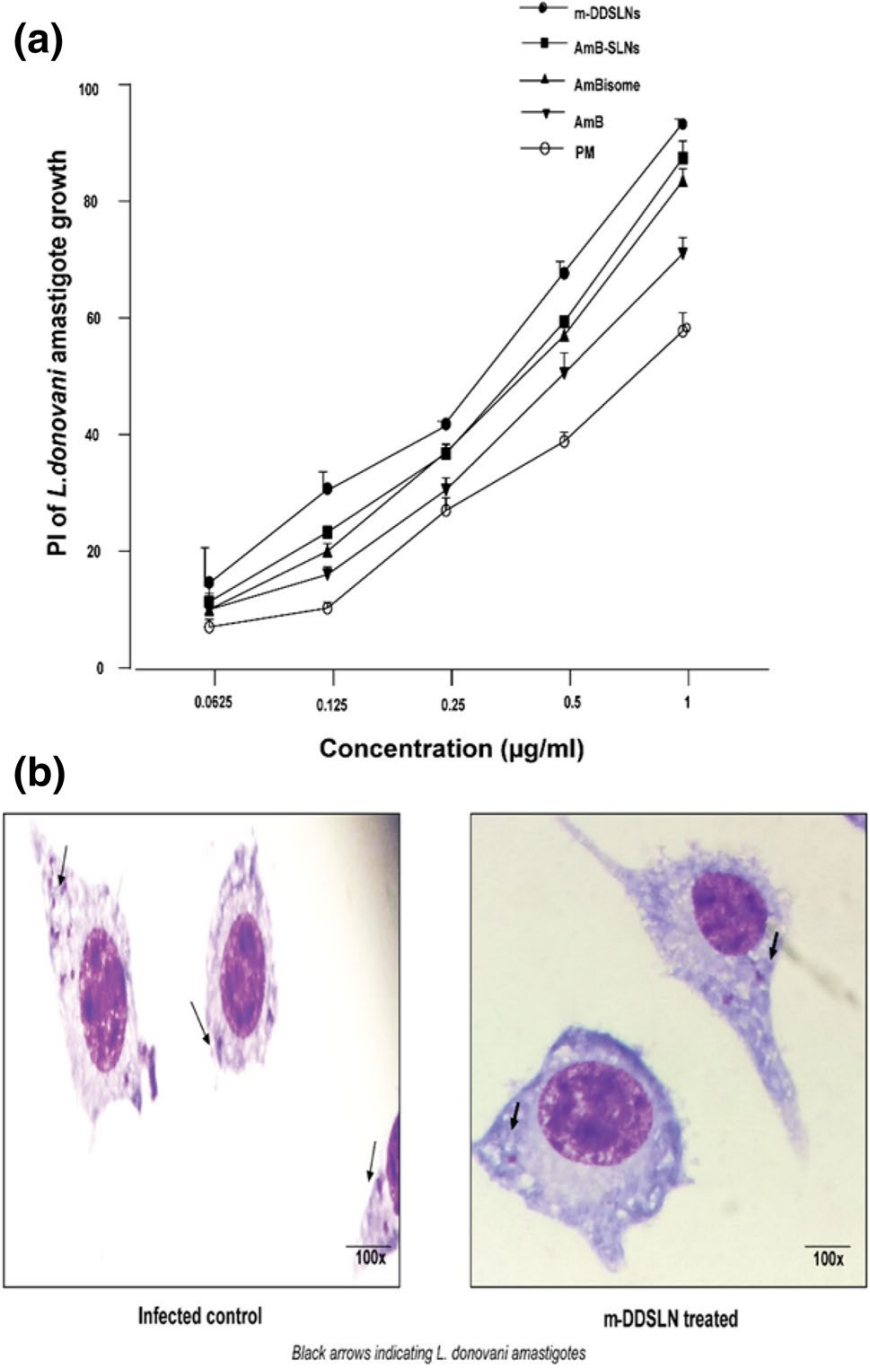
(**a**) Percentage inhibition of m-DDSLNs, AmB-SLNs, AmBisome, AmB and PM against *L. donovani*-infected J774A.1 macrophage, (**b**) microphotographs of m-DDSLNs treated and untreated *L. donovani*-infected J774A.1 macrophages. Results are presented as mean ± standard deviation (n = 3).

Inhibition of amastigote growth in intra-cellular macrophages by m-DDSLNs, AmB-SLNs, AmBisome, AmB and PM were evaluated. All samples were stained and determined the intra-cellular amastigote growth. m-DDSLNs has significantly (*P* < 0.001) reduced the intra-cellular amastigotes compared to free AmB and PM. AmB-SLNs has also shown significant (*P* < 0.01) reduction in intra-cellular amastigotes as compared to free AmB. The IC_50_ value of m-DDSLNs was 0.01328 ± 0.008598 µg/ml which was significantly (*P* < 0.001) lower than IC_50_ values of AmBisome (0.188059 ± 0.055942 µg/ml) and free AmB (0.388796 ± 0.020433 µg/ml). The IC_50_ value of mDDSLNs has 10.07-, 14.46-, 23.07- and 76.92-times more effective than AmB-SLNs, AmBisome, AmB and PM respectively. The efficient uptake by RES macrophages that act as a secondary reservoir providing extended exposure to the leishmanial cells could be the possible reason for higher efficacy. The enhanced anti-leishmanial activity against macrophages is attributed to HPCD surface modification, which favored the formulations towards enhanced macrophage internalisation^66^. m-DDSLNs (1 µg/ml) has shown maximum percentage of inhibition (96.22%) on intra-cellular amastigote growth of *L. donovani* (Fig. 6a,b). It has been reported HPCD modification of piperolactam A loaded nanoparticles has shown lesser toxicity and enhanced amastigote killing effect in both wild and resistant strains of leishmania^68^. Baek et al. have reported the enhanced cellular uptake of paclitaxel from HPCD modified paclitaxel (PTX) solid lipid nanoparticles (SLNs). HPCD was easily dissolved in the extracellular fluid, and SLNs of PTX were separately uptaken into the intracellular fluid by endocytosis^36^.

### In vivo toxicity study

**Figure 7:**
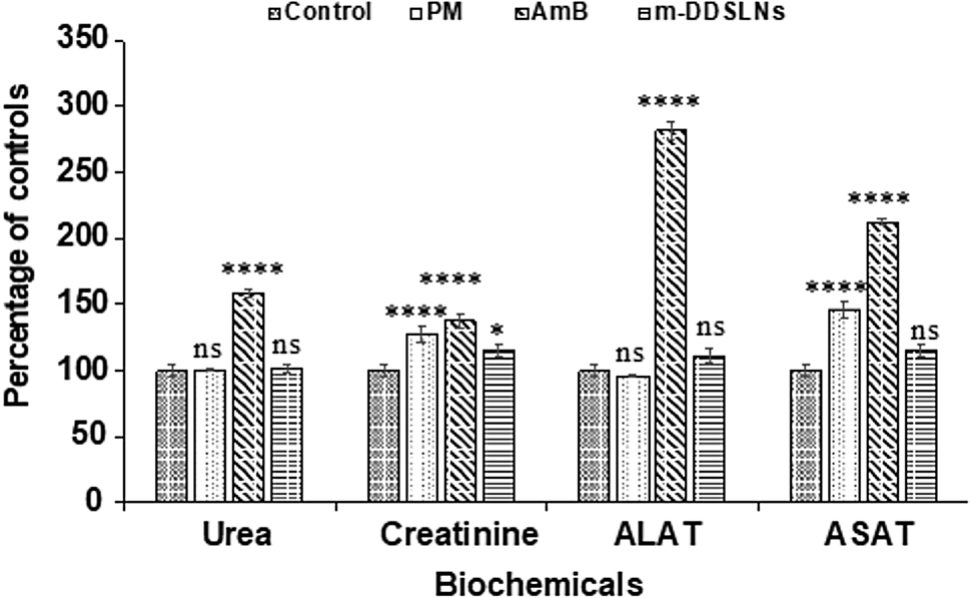
Serum biochemical analysis of AmB, PM, m-DDSLNs, aspartate aminotransferase (ASAT), alanine aminotransferase (ALAT), serum creatinine and blood urea for injection daily for 14 days (n = 3). Results are presented as the mean ± standard deviation (n = 3). Statistical significance was assessed using twoway ANOVA followed by Student's t-test (****P < 0.0001).

The major limitation of conventional AmB treatment is hepatic and renal toxicity concerns. Figure 7 represents the levels of renal (BUN and creatinine) and hepatic (SGOT, SGPT) toxicity markers in serum. A significant increase (*P* < 0.0001) in the levels of all the serum biomarkers was observed in AmB treated animal group compared to the control group. On the contrary, m-DDSLNs treated mice didn’t show any significantly increased levels of all the serum biomarkers. Our results also throw light on the safer toxicity profile of the m-DDSLNs, when administered orally without affecting the liver and kidney functions.

### In vivo anti-leishmanial activity of m-DDSLNs in *L. donovani*-infected BALB/c mice

**Figure 8:**
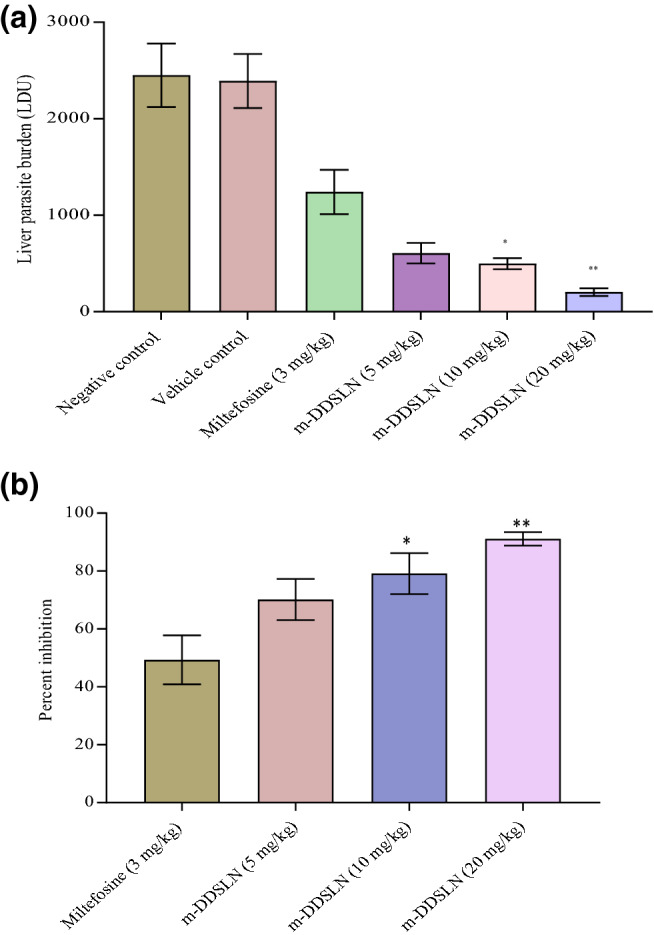
(**a**) In vivo anti-leishmanial activity of m-DDSLNs and miltefosine against *L. donovani*-infected BALB/c mice. DDSLNs (5–20 mg/kg) and miltefosine (3 mg/kg) were given orally to 7 days post infected mice for 5 consecutive days and day + 14, all the animals were sacrificed for parasitological observations. Results are presented as mean ± standard deviation (n = 4). (**b**) In vivo anti-leishmanial activity of m-DDSLNs and miltefosine against *L. donovani*-infected BALB/c mice. PI of m-DDSLNs (5–20 mg/kg) and miltefosine (3 mg/kg) were calculated as compared to vehicle treated animals. All values were expressed as mean ± standard deviation (n = 4).

m-DDSLNs (20 mg/kg × 5 days, *p.o.*) has significantly (*P* < 0.01) reduced the liver parasite burden as compared to miltefosine (3 mg/kg × 5 days, *p.o.*) in VL-infected mice. m-DDSLNs (10 mg/kg × 5 days, *p.o.*) has also shown significant (*P* < 0.05) reduction in liver parasite burden as compared to miltefosine (3 mg/kg × 5 days, *p.o.*) (Fig. 8a) Oral administration of m-DDSLNs (20 mg/kg × 5 days) has shown 91.12 ± 2.79% inhibition of the liver parasite burden, whereas, m-DDSLNs (10 mg/kg × 5 days), m-DDSLNs (5 mg/kg × 5 days) and miltefosine (3 mg/kg × 5 days) has shown 79.12 ± 7.05%, 70.14 ± 7.12% and 49.30 ± 8.47% parasite inhibition respectively against *L. donovani*-infected mice model (Fig. 8b). The enhanced efficacy of the formulation is accredited to the combinatorial cargo system with anti-leishmanial drugs. Surface modification with HPCD can enhance the oral bioavailability and tissue permeability m-DDSLNs without causing any toxic effects^69^.

## Conclusion

In this study, m-DDSLNs of AmB and PM were successfully prepared by the emulsion solvent evaporation method by using HPCD as a surface modifier for the enhancement of oral bioavailability in the treatment of VL. Formulated m-DDSLNs were characterized physico-chemically and tested for its efficacy. The prepared m-DDSLNs showed enhanced efficacy than liposomal AmB, in both in vitro and in vivo by reducing intracellular amastigote growth in *L. donovani*-infected macrophages and hepatic parasite burden in *L. donovani*-infected BALB/c mice model, respectively without causing any toxic side effects. The ability of such modified SLNs support promising utility of the delivery system as an alternative to conventional therapeutics towards infectious diseases.
